# Disease Severity in Patients Infected with *Leishmania mexicana* Relates to IL-1β

**DOI:** 10.1371/journal.pntd.0001533

**Published:** 2012-05-22

**Authors:** Edith A. Fernández-Figueroa, Claudia Rangel-Escareño, Valeria Espinosa-Mateos, Karol Carrillo-Sánchez, Norma Salaiza-Suazo, Georgina Carrada-Figueroa, Santiago March-Mifsut, Ingeborg Becker

**Affiliations:** 1 Departamento de Medicina Experimental, Facultad de Medicina, Universidad Nacional Autónoma de México, México Distrito Federal, México; 2 Instituto Nacional de Medicina Genómica, México Distrito Federal, México; 3 Secretaría de Salud del Estado de Tabasco, Universidad Juárez Autónoma de Tabasco, Tabasco, México; 4 Universidad Juárez Autónoma, de Tabasco, Tabasco, México; René Rachou Research Center, Brazil

## Abstract

*Leishmania mexicana* can cause both localized (LCL) and diffuse (DCL) cutaneous leishmaniasis, yet little is known about factors regulating disease severity in these patients. We analyzed if the disease was associated with single nucleotide polymorphisms (SNPs) in IL-1β (−511), CXCL8 (−251) and/or the inhibitor IL-1RA (+2018) in 58 Mexican mestizo patients with LCL, 6 with DCL and 123 control cases. Additionally, we analyzed the *in vitro* production of IL-1β by monocytes, the expression of this cytokine in sera of these patients, as well as the tissue distribution of IL-1β and the number of parasites in lesions of LCL and DCL patients. Our results show a significant difference in the distribution of IL-1β (−511 C/T) genotypes between patients and controls (heterozygous OR), with respect to the reference group CC, which was estimated with a value of 3.23, 95% CI = (1.2, 8.7) and p-value = 0.0167), indicating that IL-1β (−511 C/T) represents a variable influencing the risk to develop the disease in patients infected with *Leishmania mexicana*. Additionally, an increased *in vitro* production of IL-1β by monocytes and an increased serum expression of the cytokine correlated with the severity of the disease, since it was significantly higher in DCL patients heavily infected with *Leishmania mexicana*. The distribution of IL-1β in lesions also varied according to the number of parasites harbored in the tissues: in heavily infected LCL patients and in all DCL patients, the cytokine was scattered diffusely throughout the lesion. In contrast, in LCL patients with lower numbers of parasites in the lesions, IL-1β was confined to the cells. These data suggest that IL-1β possibly is a key player determining the severity of the disease in DCL patients. The analysis of polymorphisms in CXCL8 and IL-1RA showed no differences between patients with different disease severities or between patients and controls.

## Author Summary

*Leishmania mexicana* is an intracellular parasite that causes two polarly opposed diseases: One is a self-limited disease, characterized by ulcerative lesions associated with a low infectious load, as found in patients with localized cutaneous leishmaniasis (LCL). And the other pole is characterized by a progressive disease where abundant parasites spread uncontrollably throughout the skin inside heavily infected phagocytic cells, as occurs in patients with diffuse cutaneous leishmaniasis (DCL). The cause of this severe form of the disease is unknown, although the early encounter between the parasite and the inflammatory response of the host possibly plays a decisive role in the disease outcome. We here show that polymorphism in the gene encoding IL-1β (−511 C/T) represents a variable influencing the risk to develop the disease for patients infected with *Leishmania mexicana*. *In vitro* experiments showed that monocytes of DCL patients secreted significantly higher levels of the proinflammatory cytokine IL-1β as compared to LCL patients. DCL patients also had augmented levels of IL-1β in serum, and the cytokine was diffusely distributed throughout lesions, which was correlated with the numbers of parasites in the lesions. We propose that IL-1β possibly plays a key role in establishing the disease severity in patients infected with *Leishmania mexicana*.

## Introduction

*Leishmania mexicana* can cause a wide spectrum of clinical diseases, ranging from a localized cutaneous ulcer at the infection site, which is characteristic for patients with localized cutaneous leishmaniasis (LCL), to a disseminating disease, where intensely parasitized macrophages form nodules that spread throughout the skin and ultimately invade the oropharyngeal and nasal mucosae, which is characteristic for patients with diffuse cutaneous leishmaniasis (DCL). Whereas LCL patients have a cellular immune response associated with macrophage-activating cytokines such as IFN-γ, DCL patients lack an effective cellular immune response, permitting an uncontrolled replication of the parasites within macrophages and other phagocytic cells. Little is known regarding the factors involved in modulating the disease outcome; one of the possible factors are early inflammatory mediators [1]–[5]. An excessive inflammatory response can lead to increased neutrophil infiltration, which has been associated with disease progression [6], [7]. The observation that enhanced neutrophil recruitment contributes to disease susceptibility has been confirmed in experimental mouse models, which showed that an improvement in disease outcome was associated with a decrease in neutrophil immigration into the lesions [8]. One of factors responsible for neutrophil infiltration is IL-1β [9]. This cytokine also induces other innate mediators such as acute phase proteins and chemokines such as IL-6 and CXCL8 (IL-8), respectively [10].

Production of active IL-1β by monocytes is promoted by inflammasomes in response to diverse stimuli such as infections [11], [12]. NALP3, which belongs to the large family of intracellular Nod-like receptors (NLRs), associates by oligomerization with other intracellular proteins to form a complex known as the inflammasome, which converts inactive procaspase 1 to active caspase 1. This enzyme then cleaves the inactive IL-1β precursor to a secreted active IL-1β [13].

Single nucleotide polymorphisms (SNPs) of IL-1β have been associated with susceptibility towards various inflammatory diseases, such as gastric cancer [14], [15], periodontal disease [16], inflammatory bowel diseases [17] and nasal polyposis [18], among others. IL-8 (−251) has been associated with an increased risk to develop *H. pylori*-associated gastroduodenal disease [19]. Vairaktaris et al. (2005) [20] suggested that IL-8 (−251) may be a major contributor to genetic risk factor in oral cancer. IL-1RA (+2018) has been shown to be associated with a significant increase in the risk of developing fibrosing aveolitis [21].

Even though elevated levels of mRNA IL-1β have been reported in biopsies of patients with American cutaneous leishmaniasis [22], neither IL-1β (−511), CXCL8 (−251) nor IL-1RA (+2018), have been associated with disease in leishmaniasis. In this work we analyzed these SNPs in mononuclear cells of patients with different clinical forms of cutaneous leishmaniasis. Additionally, we analyzed the *in vitro* production of IL-1β by patient monocytes, the expression of IL-1β in the sera and the cytokine distribution in the cutaneous lesions of both groups of patients. We found that polymorphism in IL-1β (−511 C/T) is associated with a higher risk to contract the disease when the patients are infected with *Leishmania mexicana*. We were also able to demonstrate that patients with the more severe form of the disease that harbor a larger number of *Leishmania mexicana* parasites, show an enhanced *in vitro* production of IL-1β by monocytes, an increased serum expression of IL-1β and a diffuse distribution of IL-1β in the lesions.

The analysis of polymorphisms in CXCL8 and IL-1RA showed no differences between patients and controls (data not shown).

## Materials and Methods

### Ethical statement

This study was conducted according to the principles expressed in the Declaration of Helsinki. The study was approved by the Institutional Ethics Committee of the Medical Faculty of the National Autonomous University of Mexico (FMED/CI/RGG/013/01/2008) and guidelines established by the Mexican Health Authorities were strictly followed. All patients provided written informed consent for the collection of samples and subsequent analysis.

### Patients and controls

For the analysis of IL-1β polymorphism a total of 58 LCL patients, 6 DCL patients and 123 control cases were included. Patients were unrelated individuals and were clinically diagnosed as LCL or DCL by Giemsa-stained smears of the lesions and Montenegro skin hypersensitivity test taken at the sanitary jurisdiction office of the Cunduacán Municipality in Tabasco State, located in southeastern Mexico. The diagnosis was confirmed by an ELISA test for *Leishmania* in our laboratory. The controls had no history of the disease and were negative in the ELISA test for *Leishmania*. Both, patients and controls lived in *La Chontalpa* - a region in the state of Tabasco, Mexico, with a population of Maya ancestry mainly characterized as Mexican-mestizo. This area is endemic for leishmaniasis and patients were chosen based on the requirement that they had been locals for at least three generations so the admixture analysis could be done assuming the usual parental populations.

### Genomic DNA extraction and typing assays

Blood samples were taken from patients and controls and peripheral blood mononuclear cells (PBMC) were obtained by density-gradient centrifugation with Ficoll-Hypaque (Sigma-Aldrich St. Louis, MO, USA). Mononuclear cells were suspended in 1 mL of TRIZOL Reagent (Invitrogen Carlsbad, CA, USA), mixed and incubated for 5 min at RT. Then, 0.2 mL chloroform were added (Sigma). The resulting solution was mixed and centrifuged at 19357×g for 15 min at 4°C. The transparent phase was discarded and 0.5 mL of absolute ethanol were added (Sigma). The resulting solution was mixed and centrifuged at 19357×g for 10 min at 4°C. The supernatant was discarded and 1 mL sodium citrate 100 mM (Sigma) was added to wash the pellet, it was mixed 30 min at RT and centrifuged twice at 2151×g for 5 min at 4°C. The pellet was washed with 1 mL ethanol 75%, mixed during 20 s and centrifuged at 2151×g for 5 min at 4°C. The ethanol was dried at RT and the pellet was suspended in RNase free water.

The presence of SNPs was analyzed for IL-1β −511 (rs16944; TaqMan C_1839943_10), IL-1RA +2018 (rs419598; TaqMan C_8737990_10) and CXCL8 −251 (rs4073; TaqMan C_11748116_10) using 20 ng genomic DNA for the PCR analysis. Allelic discrimination was done using VIC and FAM fluorogenic TaqMan probes and the 5′ nuclease assay. The PCR conditions included a step at 50°C for 2 min, a polymerase activation step at 95°C for 10 min followed by 40 cycles at 95°C during 15 s and 60°C for 1 min. These assays were done in a 7900 HT Fast Real Time PCR System. The call rates we established for the analysis were 90% individually (for every sample) and for every SNP. Experimentally the call rate we found was 99% for IL-1β, 99.5% for IL-1RA and 92% for CXCL8 (IL-8).

### Statistical Analysis

For genotyping, the statistical analysis was done with Universal R software.

For every SNP in the analysis, the genotype and allele frequencies were calculated and contingency tables were produced. The Hardy Weinberg equilibrium was calculated only in the control group using the Pearson Chi-squared test. The genotype frequencies were compared among cases and controls. The homozygote, heterozygote and serological ORs were calculated with both homozygote groups of reference. The statistical significance of these was evaluated using a Chi-squared test for association with a p-value of p≤0.05 as the threshold. Since each of these SNPs was selected for biological reasons before the data was collected, multiple comparison issues were not considered appropriate. Woolf [23] confidence intervals were also calculated. Based on this, we found statistical evidence that for the IL-1β polymorphism, the heterozygote group has a higher risk for disease development than the two homozygote ones.

The statistical analysis of the data for parasite numbers, Western-blot and ELISA tests, where different numbers of patients and controls were included, was done using the Mann Whitney test and p≤0.05 was considered significant. These statistical analyses were done using the Prism 5 software (GraphPad Software, San Diego, CA, USA).

### Analysis of serum IL-1β by Western-blot

The serum expression of IL-1β was analyzed by Western-blot in 9 LCL patients, 7 DCL patients and 4 controls. Venous blood was drawn and allowed to clot at 37°C for 2 h. Serum was separated by centrifugation at 576×g for 10 min at 4°C. Sera were diluted 1∶2 with glycerol and stored at −20°C. For the Western-blot analysis, protein concentration was determined using DC Protein Assay Reagents Package (Bio-Rad Laboratories, Hercules, CA, USA) and 120 µg of serum protein were analyzed by SDS–PAGE in 15% acrylamide gels. Proteins were transferred onto Immobilon-P membranes using a semidry electroblotting apparatus. The membranes were blocked with 5% milk in Tris-buffer saline-Tween 20 (TBST: 10 mM Tris–HCl, pH 7.4, 0.15 M NaCl, and 0.05% Tween 20) for 1 h at RT. Blots were incubated with cleaved IL-1β (Asp116) polyclonal antibody (17 kDa, mature form of human IL-1β) (2021S, Cell Signaling Technology, Danvers, MA, USA) diluted in TBST with 5% BSA at 4°C overnight with shaking. Anti-rabbit IgG, HRP-linked (Cell Signaling Technology) diluted 1∶3000 in 5% non-fat dry milk was used as secondary antibody with shaking at RT for 1 h. Blots were developed using Luminata Forte Western HRP substrate (Millipore Corporation, Billerica, MA, USA) and exposed to X-ray films. The densitometric analysis was performed by recording the intensity of the bands with a MultiImage Analyzer (Alpha Innotech Corporation) based on the percentage of integrity density value (IDV).

### Monocyte purification

For the analysis of the *in-vitro* production of IL-1β by monocytes isolated from human PBMC, blood samples were taken from 5 patients with cutaneous leishmaniasis with varying degrees of disease severity which included 3 LCL patients, one DCL patient with a lesser degree of dissemination and one DCL patient who was severely infected, having numerous nodules covering the entire body surface. As for controls, blood samples from 7 healthy individuals were used, of which 3 were born and lived within the same geographical area as the LCL and DCL patients, but had been never developed the disease. The other 4 blood samples were obtained from healthy blood donors of the General Hospital of the Ministry of Health in Mexico City. Cells were separated by gradient centrifugation using Ficoll-Hypaque and mononuclear cells were isolated from the interface and washed. For the purification of monocytes, magnetically-labelled CD14 microbeads (Miltenyi Biotec, Bergisch Gladbach, Germany) were used. CD14+ monocytes were washed and plated in 24-well culture-plates.

### Cytokine measurement

The IL-1β production by non-stimulated monocytes was analyzed as follows: 1×10^6^ cells were incubated for 18 h at 37°C and 5% CO_2_ in 1 mL RPMI-1640 (Gibco, Grand Island, NY) medium, supplemented with 10% heat-inactivated FBS, endotoxin free (Gibco). The cell-free supernatants from cultures were harvested and the concentrations of the cytokine were determined by ELISA. 96-well microtiter plates (Costar, Corning, NY) were coated and sealed overnight at 4°C with assay diluent and unconjugated anti-cytokine capture antibody (88–7010 eBioscience). The wells were washed 5 times with wash buffer, blocked at RT for 1 h with assay diluent and washed again. The standard curve was performed with 2-fold serial dilutions (from 3.9 to 500 pg/mL) and samples were incubated overnight at 4°C and washed. Avidin-HRP diluted in assay diluent was added and incubated at RT for 30 min and washed 7 times with wash buffer. Each well was incubated for 15 min at RT with substrate solution and the stop solution was added. The plate was read in a μQuant spectrophotometer (BIO-TEK, Vermont, USA) at 450 nm using the KC4 v3.4 program for the analysis of samples.

### Immunohistochemistry (IHC) of tissue lesions

Skin punch biopsies (4–6 mm) were taken from the lesions of 6 DCL patients and 11 LCL patients; 8 of these LCL patients had 1 lesion ranging between 1 and 1.5 cm and 3 of the LCL patients had multiple lesions [3 to 6] ranging between 1 and 2 cm. The tissues were embedded in OCT compound and snap-frozen. They were cut into 4-µm thick slices, fixed in acetone PA (J. T. Baker) for 10 min at RT and hydrated in Tris-HCl 0.01M, NaCl 0.15M pH = 7.4. Samples were blocked for endogenous peroxidase (Peroxo-Block, Invitrogen) and for nonspecific staining (protein block solution, Abcam, Cambridge, UK). Thereafter, samples were stained with anti-IL-1β (1∶100, ab8320, Abcam) or mouse anti-*Leishmania mexicana* immune sera for 1 h at RT and secondary antibodies were used as specified by the manufacturer (mouse and rabbit specific HRP/AEC detection IHC kit, ab94705, Abcam). The slides were counterstained with Mayer's haematoxylin (Biogenex, CA, USA). Normal skin without lesions was used as a negative-control. Digital images of tissue sections were captured using a light microscope and a color AxioCam MRc5 camera (Zeiss, Germany). In order to obtain the number of parasites in lesions of LCL and DCL patients, 7 pictures of each tissue were taken with a final area corresponding to 1 mm^2^. Additionally, 6 LCL patient lesions were stained with anti-*L. mexicana* antibodies for the parasite count. These 6 biopsies had previously been taken for routine diagnostic purposes. Taken together, parasites were counted in a total number of 17 LCL patients with lesions ranging between 1 and 1.5 cm.

## Results

### Polymorphism analysis

Within the control group, all SNPs were found to be in Hardy Weinberg equilibrium (HWE) with a p-value of p = 0.0226 and for cases (including LCL and DCL patients) they were in HWE with a p-value of p = 0.024. We also calculated the HWE with cases and controls as one population with the Haplowiew 4.1 software where the HWE test failed yielding a p-value of p = 0.4998. The comparison of the genotype frequencies between LCL patients and controls for the IL-1β polymorphism showed that the homozygote frequencies were higher in the controls for both of the alleles, whereas the heterozygote frequency was higher in LCL patients compared to controls (60.3% y 38.2%, respectively) see TABLE 1. This suggests that we may have a risk associated to the development of the disease for the heterozygous genotype as opposed to a single allele risk association. As mentioned above, the odd ratios resulting of comparing the heterozygote C/T to the homozygote groups were found to be significant. The OR that compared it to the homozygote C/C was 3.23 [p-value = 0.0167, 95% CI = (1.2,8.7)]; the OR that had the homozygote T/T as reference group was 2.19 [p-value = 0.0274, 95% CI = (1.08,4.4)]. All other ORs calculated are shown in TABLE 2. In contrast, the results for CXCL8 −251 and IL-1RA +2018 were not statistically significant (data not shown).

**Table 1:** Distribution of genotypic and allelic frequencies for IL-1β −511 (C/T) polymorphism in cases and controls.

Subjects	IL-1β (−511 C/T) genotypes number (%)			Allele frequencies in %		Gender % (M/F^1^)	Age (years)
	CC	CT	TT	C	T		
Cases	6 (10.3)	35 (60.3)	17 (29.4)	40.5	59.5	M = 29, F = 26	11–64
Controls	26 (21.1)	47 (38.2)	50 (40.7)	40.2	59.8	M = 35, F = 66	7–88

1 M = Male, F = Femele, (3 cases and 22 controls, gender unknown). All patients were diagnosed by Giemsa-stained smears of the lesions, Montenegro skin hypersensitivity test and/or ELISA against Leishmania.

**Table 2:** Statistical analysis of IL-1β −511 SNP.

Group	Reference group	OR	CI	x^2^	p-value
CT	CC	3.23	(1.2,8.7)	5.72	0.016
TT	CC	1.47	(0.5,4.1)	0.53	0.465
CT+TT	CC	2.32	(0.9,6.0)	3.15	0.075
CT	TT	2.19	(1.08,4.4)	4.86	0.027
CC	TT	0.68	(0.2,1.9)	0.53	0.465
CT+TT	TT	1.65	(0.8,3.2)	2.17	0.140

Since the sample size of DCL patients is only 6, it is questionable to draw any conclusions from this sample. As an exploratory step for further research, we repeated the procedure above using all leishmaniasis patients (58 LCL and 6 DCL) as the case group. Results for IL-1β were again statistically significant: OR = 3.7 when the heterozygote C/T is compared to the homozygote C/C [p = 0.006, 95% CI = (1.38,9.85)] and OR = 2.4 when the heterozygote C/T is compared to the homozygote T/T [p = 0.012, 95% CI = (1.19,4.6)]. There seems to be no change in the conclusions drawn for the LCL patients. However, this might be due entirely to the sample size of the DCL group. No analysis was performed using only DCL patients as cases, due to the small sample size. Based on the discussion of Sasieni (1997) [24], we decided not to use the allelic OR. The second-order analysis (gene-gene association) of the samples used in this study showed no significant results (data not shown).

### Serum IL-1β analysis by Western-blot

**Figure 1:**
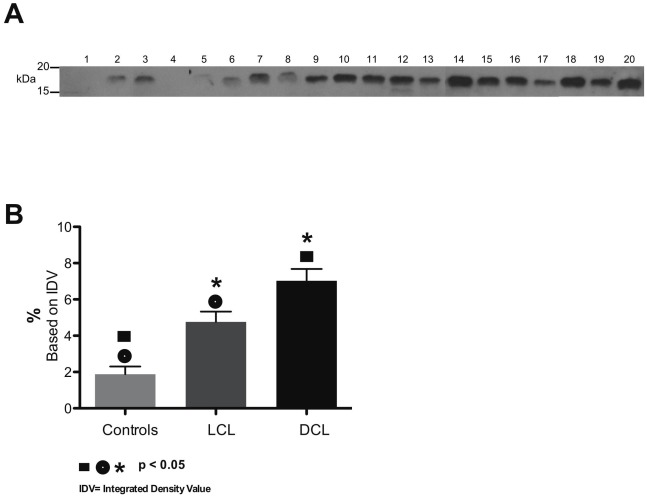
IL-1β expression in serum of patients with cutaneous leishmaniasis. (A) Western blot of mature IL-1β (17 kDa) in 4 controls (1–4), 9 LCL patients (5–13) and 7 DCL patients (14–20). (B) Graph of percentage intensity of IL-1β bands with statistically significant differences between controls *vs* LCL patients, controls *vs* DCL patients and LCL *vs* DCL patients. (Identical symbols above different bars are statistically significant: p<0.05).

The analysis of IL-1β expression in sera of LCL and DCL patients showed a different expression between both groups of patients. LCL patients showed a lower expression of this cytokine (Figure 1A, lanes 5–13) and DCL patients showed enhanced expression of IL-1β (Figure 1A, lanes 14–20). In contrast to patients with leishmaniasis, controls showed only minimal amounts of IL-1β (Figure 1A, lanes 1–4). A statistically significant difference was found (Figure 1B) when comparing IL-1β expression in sera from LCL patients *vs* controls (p = 0.01), DLC patients *vs* controls (p = 0.0052) and when comparing DCL patients *vs* LCL patients (p = 0.019).

### Cytokine production by monocytes

**Figure 2:**
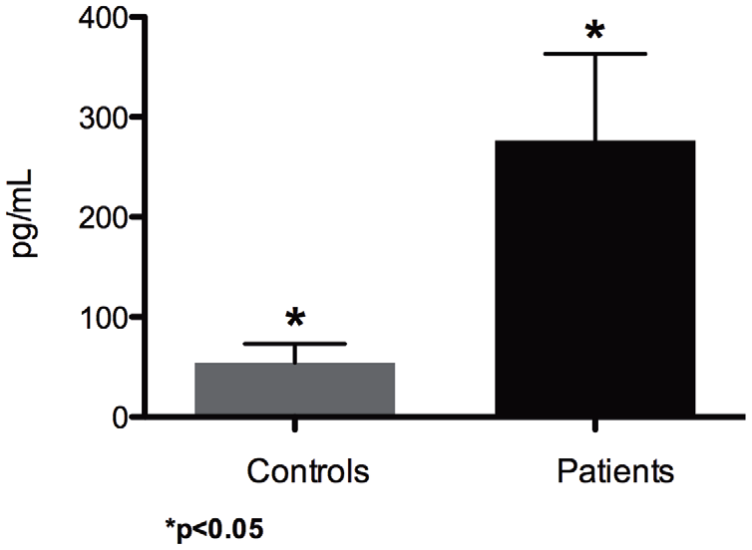
Analysis of IL-1β cytokine production by non-stimulated monocytes purified with anti-CD14 magnetic beads from PBMC of control subjects and patients with cutaneous leishmaniasis after 18 hours *in vitro* culture. Mean values obtained from 7 healthy control subjects and 5 patients with leishmaniasis. (Identical symbols above two different bars are statistically significant: p<0.05).

The analysis of IL-1β production revealed that non-stimulated monocytes from patients with cutaneous leishmaniasis had a significant increase of their production of IL-1β when compared with healthy controls (p = 0.015) (Figure 2). The individual analysis of the IL-1β production by monocytes from patients showed that the degree of IL-1β production could be related to the severity of the disease, since it was highest in patients with DCL (478 pg/mL in the DCL patient with the more severe form and 423 pg/mL in the patient with the less severe form), as compared to LCL patients in whom the production of IL-1β was between 50 to 336 pg/mL.

### Immunohistochemistry

**Figure 3:**
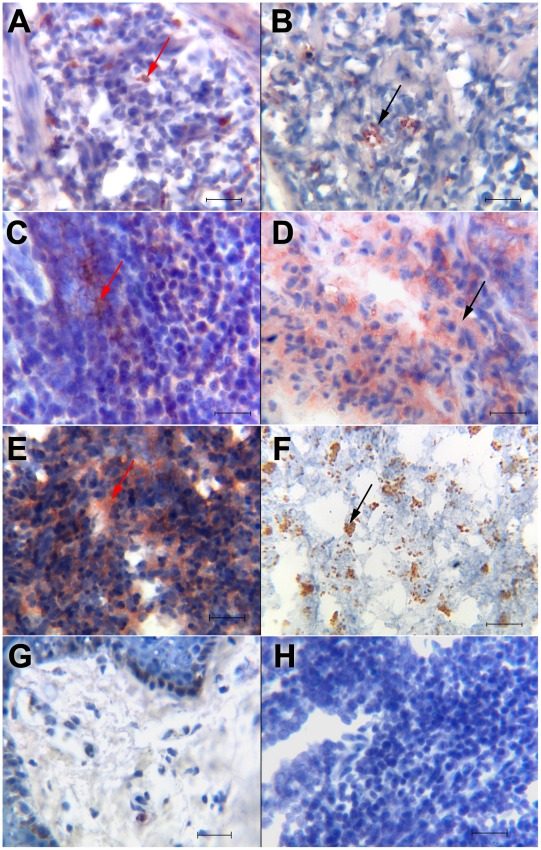
Immunohistochemistry for IL-1β and *Leishmania mexicana* staining in lesions of patients with cutaneous leishmaniasis. (A) IL-1β staining on cells of LCL patient with single small lesion; (B) small clusters of *Leishmania* parasites in LCL patient with small ulcer; (C) diffuse distribution of IL-1β in LCL patient with abundant ulcers; (D) disintegrated *Leishmania* in LCL patient with abundant ulcers; (E) diffuse distribution of IL-1β in DCL patient; (F) clusters with abundant intact *Leishmania* parasites in DCL patient. Red arrows show IL-1β^+^ staining and black arrows show *L. mexicana* staining. (G) Normal skin was used as negative control for IL-1β immunostaining. (H) Control staining with secondary antibody. All sections were counterstained with haematoxylin. (A–H) scale bar = 50 µm. Immunostaining in tissue sections was visualized at a magnification of 400×. We show a representative result of different types of lesions within each group: LCL patients with one small ulcer: (n = 8 for IL-1β staining and n = 17 for *L. mexicana* staining) (A and B); LCL patients with various ulcers (n = 3) (C and D); DCL patients (n = 6) (E and F).

The analysis of IL-1β in lesions of LCL and DCL patients showed that the cytokine distribution varied between both groups of patients: in LCL lesions, we observed two possible patterns of IL-1β expression, according to parasite numbers in the lesions. In one group (Figure 3A) the cytokine was localized on the cell surface, showing an intense stain. This type of stain was found in lesions of LCL patients that harbored few parasites (Figure 3B). The second group of LCL patients showed a diffuse distribution pattern of IL-1β staining (Figure 3C), which correlated with the diffuse staining of abundant remnants of parasites (Figure 3D). It is noteworthy that the first group of LCL patients only had one small ulcer, less than 1 cm in diameter, whereas the second group of LCL patients had 3 to 6 active lesions which varied between 1 and 2 cm in diameter. In contrast, all DCL patients showed a diffuse distribution of IL-1β throughout the lesions (Figure 3E) all of which were also heavily infected with intact *Leishmania mexicana* parasites (Figure 3F).

These results show that the characteristics of the distribution of IL-1β in the tissue varies according to parasite numbers: LCL patients with few parasites in their lesions only show cell membrane staining, whereas all DCL patients, with abundant intact parasites in their lesions as well as some LCL patients with abundant destroyed *Leishmania* parasites, show a diffuse pattern of IL-1β staining.

**Figure 4:**
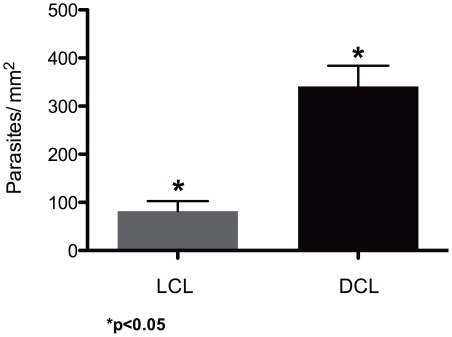
Statistical analysis of the number of parasites/mm^2^ in lesions between LCL and DCL patients. Mean values were obtained from 17 LCL patients and 6 DCL patients with leishmaniasis. (Identical symbols above two different bars are statistically significant: p<0.05).

The number of IL-1β positive cells in the tissues was different for each patient (18 to 410 cells) and did not have any correlation with the time of evolution of the disease nor with the numbers parasites (data not shown). The number of parasites was significantly different in the lesions of LCL and DCL patients (p = 0.0003) (Figure 4).

## Discussion

The aim of the present study was to determine single nucleotide polymorphisms of IL-1β (−511), CXCL8 (−251) and IL-1RA (+2018) in patients with cutaneous leishmaniasis infected with *Leishmania mexicana*. We evaluated two groups of persons (cases and controls) that lived in the same endemic region. This study for the first time demonstrated polymorphism in the gene IL-1β −511 in Mexican-mestizo patients from Tabasco, a state with high prevalence of patients with cutaneous leishmaniasis. Our results show that 90% of the individuals infected with *Leishmania mexicana* had, either a heterozygote genotype (CT), or were homozygous for the minor allele (TT). Of these, 60% had the heterozygous genotype (CT), as compared to only 38% for the healthy controls. These data suggest that the presence of this polymorphism in a heterozygous genotype may favor disease development in patients infected with *Leishmania mexicana*.

Disease susceptibility or resistance has been correlated to genetic variations [25]. Specifically, polymorphism in the gene for IL-1β (−511) has been associated with increased inflammation [26]. This cytokine activates the vascular endothelium, enhancing adhesion molecule expression, which, in combination with local vasodilatation, slows blood flow, permitting the tethering of neutrophils to the vessel wall. Locally released CXCL8 acts as an activator and chemoattractant for neutrophils [27], which can produce significant damage due to the release of pro-inflammatory granules and enzymes [28]. Inflammation has been shown to be a hallmark in cutaneous leishmaniasis and various polymorphisms, related to increased or extended inflammation, have been associated with the disease [29]–[31]. Neutrophils have been shown to play an important role in leishmaniasis. They are decisive for the early establishment of the disease following delivery of parasites by the bite of the sand fly [6] and their enhanced recruitment has been shown to contribute to disease susceptibility [8]. The facilitating role of neutrophils has been associated with their capacity to phagocytose the parasites and rapidly transport them from the infection site, thus avoiding the toxic effects of complement and the local immune responses [7]. The inflammatory response aids *Leishmania*-infected phagocytic cells to enter lymphatic vessels, favoring parasite distribution towards peripheral tissues [32]. It is therefore tempting to speculate that augmented IL-1β production possibly facilitates disease progression in patients with DCL by enhancing the inflammatory response and thereby aiding parasite dissemination. Thus we suggest that IL-1β (−511 C/T) genotype heightens the risk of developing the disease due to the fact that this polymorphism is located in the promoter region of the IL-1β gene, which has been related to enhanced cytokine production and enhanced inflammatory disease. We propose that patients with this genotype are likely to be associated with an enhanced production of the pro-inflammatory cytokine IL-1β, when the patient is infected with *Leishmania mexicana*. The enhanced *in vitro* production of IL-1β by monocytes, and the augmented expression of this cytokine in sera of patients heavily infected with *Leishmania mexicana*, possibly strengthen the importance of IL-1β as a factor to develop the more severe form of the disease, although we cannot rule out that the enhanced IL-1β production is a consequence, rather than a cause, of the more severe form of the disease. Our data are in accordance with the literature [33] regarding the presence of the pro-inflammatory cytokine IL-1β in severe tissue lesions. Additionally, disease exacerbation has also been related to IL-1β in the BALB/c mouse model [34]. Furthermore it has been reported that IL-1β^−/−^C57BL/6 mice, infected with *Leishmania major*, were resistant to experimental cutaneous leishmaniasis [35].

The enhanced production of IL-1β by monocytes and serum of patients with the more severe form of the disease led us to analyze the cytokine in tissue lesions of patients with varying disease severity. We found that the distribution of IL-1β varied in tissue lesions in accordance with the numbers of parasites present in those lesions. Thus, in heavily infected lesions of some of the patients with LCL and in all of the DCL patients, the secreted IL-1β was found diffusely distributed within the lesions, whereas in LCL patients with a lower number of parasites, IL-1β was found to be within the cells. To the best of our knowledge, this differential distribution of IL-1β, according to varying degrees of inflammatory lesions, has not been described in the literature. IL-1β production can be induced by microbial products via TLR ligands. Our group has previously shown that *Leishmania mexicana* lypophosphoglycan (LPG) is a TLR2 ligand, leading to cell activation [36]. It is thus feasible, that the enhanced presence of the parasite relates to an increased IL-1β production. The differential distribution could be related to the possible presence of an alternative (noncaspase-1) mechanism of generating active forms of IL-1β extracellularly in tissues. This alternative activating pathway has been reported for a number of molecules including serine protease-3 released by infiltrating neutrophils, as well as for other proteases such as elastase, matrix metalloprotease 9 and granzyme A released from cytotoxic T cells and mast cell chymase, which can process the IL-1β precursor into an active cytokine [37], [38]. It is noteworthy that neutrophils secrete serine protease-3 together with extracellular traps, which have been shown to be released when the cells are stimulated with *Leishmania* or LPG [39], [40]. The overall consequence of the diffusely distributed IL-1β in tissue lesions that are heavily infected with *Leishmania mexicana* is not clear, since in LCL patients IL-1β was associated with parasite destruction, whereas in DCL patients the parasite was not destroyed, despite the equal distribution of IL-1β. It is therefore tempting to speculate that IL-1β is not directly involved in parasite killing, but rather in aiding the mobility of phagocytosed *Leishmania* to be transported outside of the lesions. In DCL patients with impaired leishmanicidal capacities, IL-1β possibly aids parasite distribution by enhancing the inflammatory response. Thus, enhanced IL-1β production and distribution seems more critical in patients that have underlying problems limiting their leishmanicidal capacity, such as in patients with DCL. These results open a new perspective regarding the dynamics of IL-1β release in tissues heavily infected with *Leishmania mexicana*.

In conclusion, our data show that leishmaniasis patients with IL-1β polymorphism have a heightened risk to develop the disease. Additionally we show that patients with a more severe form of the disease show an enhanced IL-1β production. Yet it remains to be established if the polymorphism relates directly with disease severity in patients infected with *Leishmania mexicana*. We propose that this might be the case, based on reports that the polymorphism is located in the promoter region of the IL-1β gene which has been shown to lead to enhanced IL-1β production [26].

Our data help shed new light on the genes involved in the disease outcome. Further studies aimed at analyzing allelic and genotypic distributions in the Mexican population will help clarify the differences we observed between cases and controls in polymorphisms implicated in susceptibility to leishmaniasis in our population. Only a few genes that determine susceptibility in the complex relationship between the parasite and the host immune response have been identified [41]. The analysis of IL-1β and other proteins that participate in inflammation like caspase-1 and -5 or NALP3, in a larger group of patients, could possibly help define to what extent polymorphisms and enhanced production of IL-1β contribute to various clinical forms of leishmaniasis.

Accession links for numbers/ID numbers for genes and proteins mentioned in the text:

IL-1β:

Protein: http://www.uniprot.org/uniprot/P01584

Gene: http://www.ncbi.nlm.nih.gov/protein/NP_000567.1

(CXCL8) IL-8:

Protein: http://www.uniprot.org/uniprot/P10145

Gene: http://www.ncbi.nlm.nih.gov/protein/AAH13615.1

IL-1RA:

Protein: http://www.uniprot.org/uniprot/P18510

Gene: http://www.ncbi.nlm.nih.gov/protein/CAA36262.1
